# The importance of bone marrow infiltration patterns in multiple myeloma seen on magnetic resonance imaging—Case report and imaging perspective

**DOI:** 10.1002/ccr3.6452

**Published:** 2022-10-17

**Authors:** Dan Costachescu, Hortensia Ionita

**Affiliations:** ^1^ Radiology Department University of Medicine and Pharmacy ‘Victor Babes’ Timisoara Romania; ^2^ Haematology Department University of Medicine and Pharmacy ‘Victor Babes’ Timisoara Romania

**Keywords:** “salt and pepper”, MRI, non‐secretory multiple myeloma

## Abstract

Non‐secretory multiple myeloma (NSMM) is an extremely rare variant of multiple myeloma (MM) and accounts for a maximum of 5% of all myeloma cases. This variant of MM usually represents a diagnostic challenge to the clinician because of the absence of detectable monoclonal immunoglobulin on serum or urine electrophoresis. We present the case of a 34‐year‐old Caucasian male who presented to the emergency department with pain in the lumbar area secondary to a fall and who was eventually diagnosed with non‐secretory multiple myeloma after the radiologist initially pointed out a discrete “salt and pepper” infiltration of the spine seen on magnetic resonance imaging (MRI) although the spine computed tomography (CT) performed initially showed no suspicious lesions for malignancy. The final diagnosis was obtained after a positive bone marrow biopsy together with the presence of malignant lesions seen on the spine MRI. This case points out the importance of different bone marrow involvement patterns seen on MRI and other useful sequences the radiologist could use to better discriminate between normal marrow reconversion and malignant infiltration.

## Graphical abstract

We describe this case report to raise awareness among medical practitioners and radiologists of the extreme importance of subtle bone marrow infiltration patterns seen on MRI, which could help diagnose multiple myeloma at an early stage.

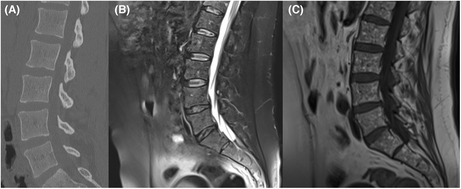

## 1 INTRODUCTION

Multiple myeloma (MM) is a plasma cell dyscrasia that is associated with the clonal proliferation of plasma cells. Non‐secretory multiple myeloma (NSMM) on the contrary is a rare variant of MM which accounts for a maximum of 5% of all myeloma cases and is even much rarer in young people.^1^ The definition of NSMM is that of symptomatic myeloma without the existence of detectable monoclonal immunoglobulin on serum or urine electrophoresis.

Both entities have the same clinical and imaging features. However, in the case of NSSM, the plasma cells fail to secrete an immunoglobulin, and therefore, both the serum and urine electrophoresis are normal.^2^ The diagnosis of these patients is achieved through bone marrow biopsy and immunohistochemistry.

The use of MRI in the diagnosis and staging of MM has increased within the last decades, MRI being more sensitive than computed tomography and X‐rays in the detection of focal MM lesions, as well as subtle medullary infiltration.^3^

We present the case of a patient with non‐secretory multiple myeloma that was diagnosed at an early stage, before end‐organ damage, thanks to the extreme sensitivity of MRI in depicting marrow infiltration, changes that in numerous instances are visible before any clinical or serological abnormalities are observed.

## 2 CASE PRESENTATION

**FIGURE 1:**
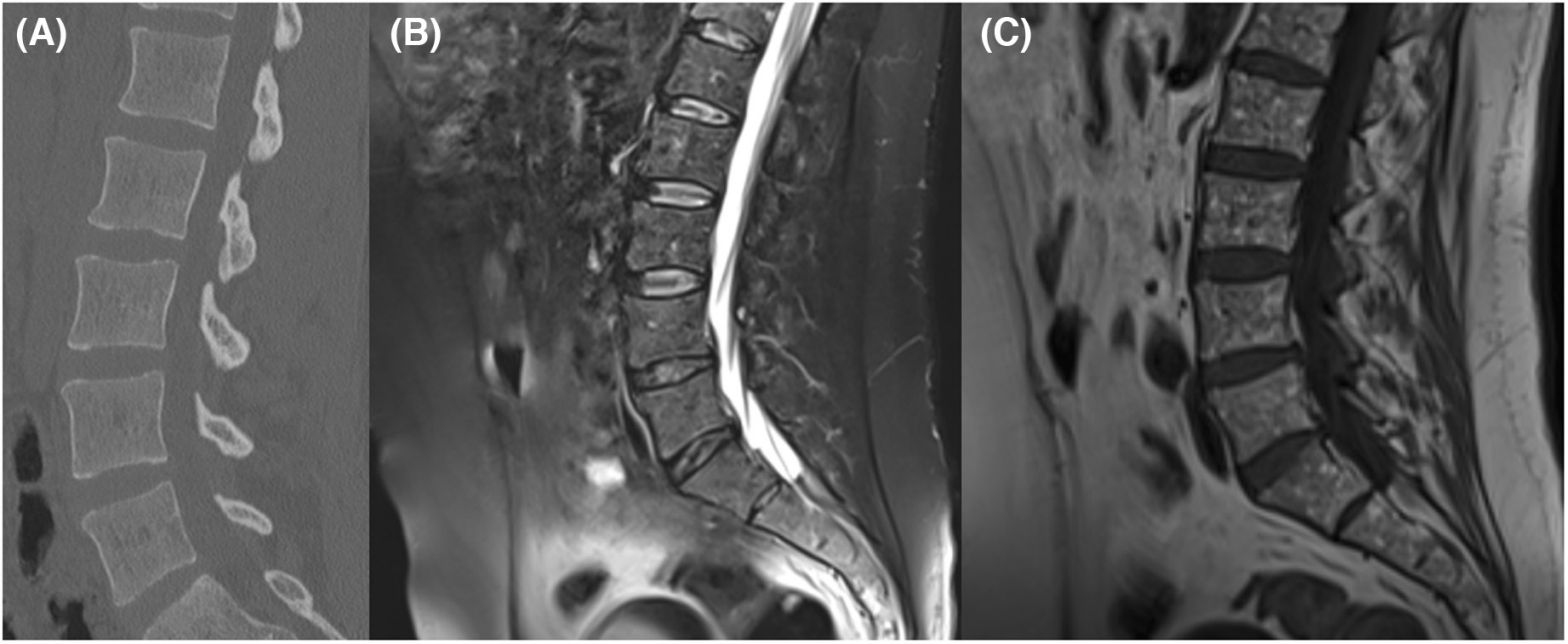
(A) Normal lumbar spine CT in the emergency department; (B) (Sagittal T2 fat saturated) and (C) (Sagittal T1)—lumbar spine MRI with no post‐traumatic changes but with a “salt and pepper” pattern seen on the sagittal T1 sequence.

A 34‐year‐old male patient presented to the Emergency Department of the Regional Hospital, complaining of lumbar back pain after a fall from a 2‐m height. The lumbar spine CT was normal (Figure 1A), demonstrating no acute pathology. The lumbar pain was persistent for the next week, and after an orthopedic examination, the patient was referred for a whole‐spine MRI; there was no bone oedema or other post‐traumatic changes (Figure 1B), but the radiologist pointed out a discrete “salt and pepper” infiltration (Figure 1C) of the bone marrow and recommended a hematological examination, raising the suspicion of a possible bone marrow infiltration in the context of multiple myeloma.

After extensive laboratory results, nothing but discrete anemia was noted; there was no detectable monoclonal immunoglobulin on serum or urine electrophoresis (Table 1).

**TABLE 1:** Laboratory results.

Parameter	Case	Normal range
Hemoglobin	12.6 g/dl	13–18 g/dl
CRP	6.53 mg/L	0–5 mg/L
ALT	25 UI/L	0–52 UI/L
AST	27 UI/L	6–35 UI/L
LDH	233 UI/L	122–249 UI/L
Creatinine	8.44 mg/L	7–12.6 mg/L
Ca++	90 mg/L	85–110 mg/L
24 h urinary protein	52.1 mg/24 h	<500 mg/24 h
Total protein	61.4 g/L	54–75 g/L
IgG	5.4 g/L	5.3–19.22 g/L
IgM	0.89 g/L	0.20–2.30 g/L
IgD	45.1 mg/L	7.4–131 mg/L
Free light chains Kappa‐serum	5.64 mg/L	3.20–18.40 mg/L
Free light chains Lambda‐serum	6.71 mg/L	6.62–25.20 mg/L
Kappa/Lambda Free light chains ratio	0.84	0.23–1.64

**FIGURE 2:**
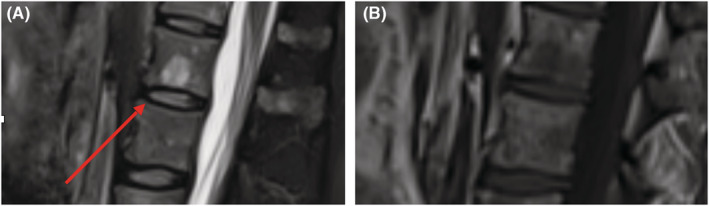
(A) Sagittal T2 fat saturated—nodular infiltration of the marrow (red arrow); (B) Sagittal T1 showing diffuse “salt and pepper” infiltration.

The patient continued to complain of worsening lumbar pain for the next 2 weeks, and another MRI was performed; this time the presence of a new nodular infiltration of the spine at the level of the L1 vertebral body (measuring 16 mm; Figure 2A) was observed, as well as the enlargement of several other nodular lesions (measuring between 5 mm and 10 mm) previously seen within the “salt and pepper” infiltration at the level of the lumbar spine. The lesions showed typical MRI characteristics of malignancy (T1 hypointense, T2 fat saturated hyperintense, with no signal drop on out‐phase images). After a multidisciplinary team meeting (MDT), the medical staff decided to perform a marrow biopsy which revealed 65% medullary plasmacytosis; the patient met the criteria for MM diagnosis according to the International Myeloma Working Group (IMWG) guidelines^4^ (Clonal bone marrow plasma cells ≥10% as well as a MM defining event; in our case, the MM defining event was the presence of >1 focal bone lesions seen on MRI with a diameter of minimum 5 mm); moreover, the patient presented all the criteria necessary (seen in Table 2) to be diagnosed with non‐secretory multiple myeloma.

**TABLE 2:** Criteria for non‐secretory multiple myeloma.^5^

Clonal plasma cells(biopsy‐proven)
<0.5 mg/dl serum protein by serum protein electrophoresis
<200 mg/24 h of light chain proteinuria by urine protein electrophoresis
Free light chain measurement unquantifiable
Positive “CRAB” criteria for symptomatic myeloma

Treatment was immediately initiated with a bortezomib‐cyclophosphamide‐dexamethasone (VCD) chemotherapy protocol for up to 8 cycles, every 21 days. The treatment was well tolerated with minimal side effects, mainly fatigue, headaches, and nausea. All the side effects responded well to symptomatic drugs. The patient is currently in complete remission with normal laboratory results except for slightly elevated AST (44 UI/L) and ALT (56 UI/L) levels which were considered secondary to therapeutic hepatotoxicity.

## 3 DISCUSSION

NSMM is a rare type of MM and is defined by the absence of detectable M protein in serum and urine. Two different subtypes of NSMM have been described. In the first subtype, the plasma cells produce immunoglobulins but are unable to release them out of the cell, probably due to decreased permeability, deficiency, or damage of intracellular light chains. This variant of NSMM is known as the “producer” type. The second form is the “non‐producer” type, in which immunoglobulin cannot be produced by plasma cells.^6^

The diagnosis of multiple myeloma is based on the presence of bone marrow plasmacytosis of >10%, monoclonal protein in serum and/or urine, as well as myeloma‐related organ dysfunction (CRAB criteria).^7^

Due to the absence of M protein in serum and urine, our patient did not meet the criteria for the diagnosis of MM but was labeled as an NSMM after the second MRI scan revealed a new bone lesion at the level of the L1 vertebral body, several other enlarged lesions (between 5 and 10 mm) within the “salt and pepper” marrow infiltration and confirmed by a positive bone marrow biopsy for plasmacytosis.

This case emphasizes the importance of modern imaging (MRI) in the diagnosis of a rare form of multiple myeloma,^8^ and the discussion is focused on the types of bone marrow infiltration in MM seen on MRI.

Computed tomography (CT) is a sensitive method for the diagnosis of lytic lesions, but it is well known that patients with multiple myeloma present bone marrow infiltration before any lytic lesions can be seen on a CT scan,^9^ a fact that was very well observed even with the presented case.

Modern imaging methods like MRI detect discrete marrow infiltration patterns before lytic lesions are detected on computed tomography and sometimes before the onset of any symptoms.^10^

**FIGURE 3:**
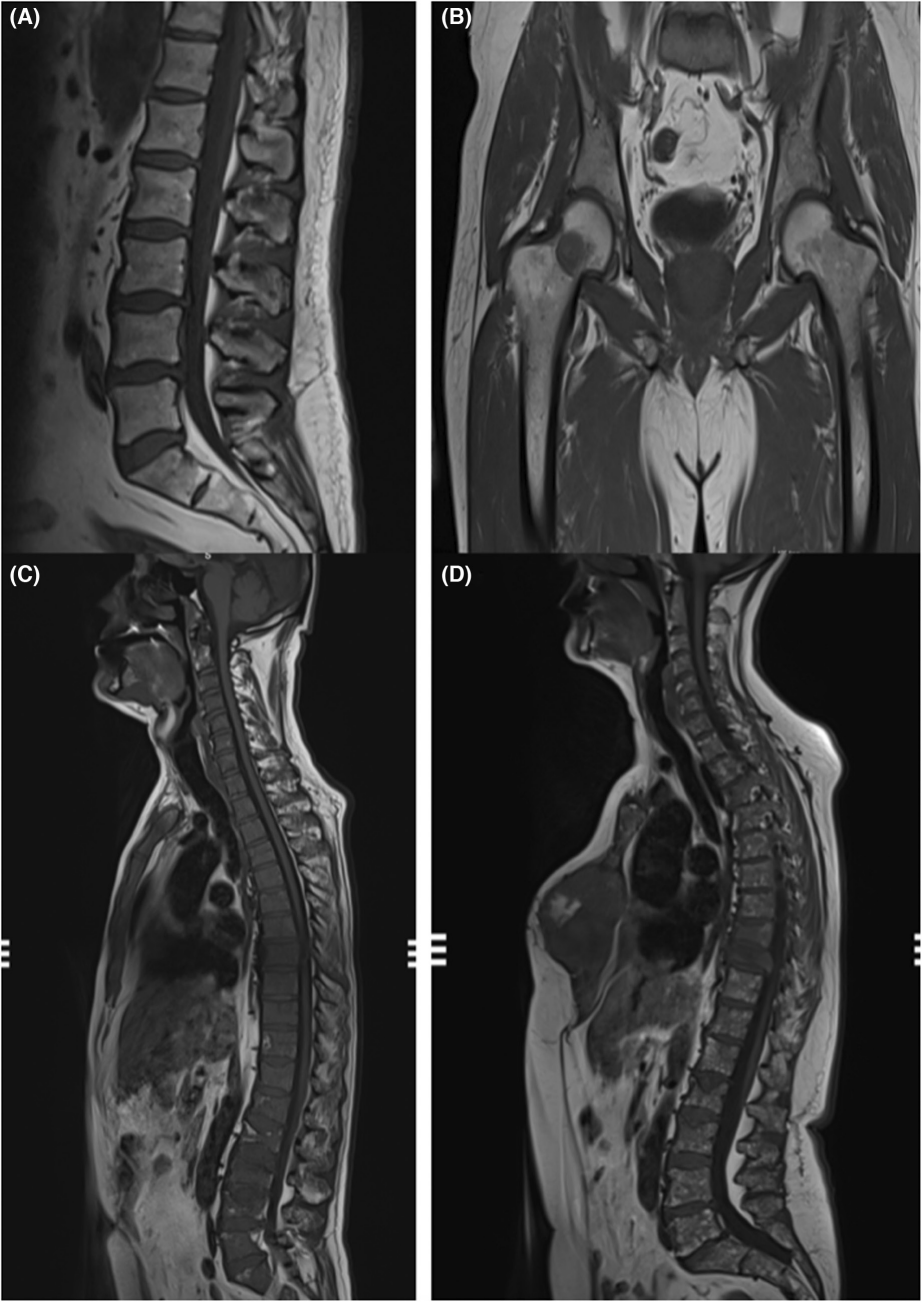
(A) Normal‐appearing bone marrow, hyperintense on T1; (B) focal nodular infiltration of the right femur; (C) diffuse infiltration of the bone marrow; (D) “salt and pepper” infiltration.

On MR imaging, five different patterns of bone marrow infiltration in multiple myeloma were described. These patterns include a normal‐appearing marrow, focal infiltration, diffuse infiltration, and “salt‐and‐pepper”; the fifth pattern is a combination of focal and diffuse infiltration^11^, ^12^ (Figure 3).

The “salt and pepper” infiltration of the bone marrow is seen in only about 3% of MM patients,^13^ thus being sometimes misleading for the inexperienced radiologists.

If in doubt, chemical‐shift imaging is a fast and easy way to discriminate between normal bone marrow and malignant infiltration, and therefore, it should be included in routine MRI examinations of patients with suspected marrow infiltration.^14^ Normal vertebrae contain a high amount of fat, and therefore, they show a loss of signal on out‐phase images. In benign vertebral lesions or bone‐marrow reconversion, there is a signal drop on out‐phase images because of the high amount of fat present. In malignant marrow diseases, there is almost complete marrow replacement with tumor cells/plasmacytes resulting in no signal drop on the out‐phase images.^14^

Another fast MRI technique to help differentiate malignant marrow lesions from benign marrow reconversion is the “functional” diffusion‐weighted (DWI) sequence and the corresponding apparent diffusion coefficient(ADC). ADC values in active myeloma are significantly higher than normal red or yellow marrow,^15^ indicating the use of diffusion‐weighted MRI to differentiate undoubtedly between normal and pathologic bone marrow.

In the case presented, the first spine MRI scan was performed in a private imaging clinic with a routine scanning protocol that included only T1, T2, and T2 fat saturation sequences; that is why the radiology report only pointed out the discrete “salt and pepper” pattern without a certain diagnosis of multiple myeloma infiltration. If further scan sequences like chemical shift imaging or DWI were performed, the radiologist's report would have been certainly more precise and the bone biopsy requested earlier.

## 4 CONCLUSION

Multiple myeloma (MM) is a plasma cell dyscrasia associated with the clonal proliferation of a defective plasma clone. Non‐secretory multiple myeloma (NSMM) is a rare variant of MM and accounts for less than 5% of all MM cases and often poses diagnostic difficulties. We present the case of a patient with NSMM who was diagnosed after a series of 2 spinal MRIs which showed bone marrow infiltration suggestive of MM (“salt and pepper” pattern). This case emphasis the need for different MRI sequences, to better discriminate between normal marrow reconversion and malignant infiltration.

## AUTHOR CONTRIBUTIONS

DC was involved in literature research and manuscript writing. HI was involved in critical feedback and manuscript revisions.

## FUNDING INFORMATION

No funding was received.

## CONFLICT OF INTEREST

The authors declare that there is no conflict of interest.

## CONSENT

Written informed consent was obtained from the patient for the publication of this case report.

## Data Availability

Other desired data and material relevant to our case report are available on request.
